# Medical News and Miscellany

**Published:** 1894-07

**Authors:** 


### Medical News and Miscellany.

---

**Dr. Swearingen** and his family are camping at San Marcos during the Chautauqua season.

---

**The Refractionist** is the name of a new journal, "intended to be the exponent of the rəfraction world." We have received a copy. It is published monthly at Boston, edited by F. F. Whittier, A. M., M. D., Professor of Clinical Ophthalmology, College of Physicians and Surgeons, etc., etc., with associate editors. Price $2 a year.

---

**Organize.**—In another place will he found a call by the physicians of Decatur, Bowie and other towns, for a meeting to organize another district medical society. This is encouraging. We pointed out, in a recent issue, the causes operating to prevent the State Association ever becoming much larger than it is at present and has been for the past ten years. It can never unify the profession, from the very nature of things; the defect is in its organization; it can never hold together those who join; the State is too large—the physicians too numerous for a society by membership; and in our next we propose to offer a few suggestions looking to making it an association of delegates—every county, or every district, at least, being organized to send representatives,—pretty much as our political organizations are operated.

---

**Death of Dr. B. A. Pope.**——Dr. Boling A. Pope, of Dallas, died in that city at 2:30 a. m., July 4th inst. Dr. Pope was born in Washington county, Georgia, in 1829, and was 65 years old. He graduated in medicine in Paris, Heidelberg and Berlin before the war and settled in New York, where he became connected with the College of Physicians and Surgeons. When the war broke out he resigned and came South, casting his fortunes with his native State. He was a Confederate surgeon and had charge of a Georgia hospital. Settled in New Orleans after the war and did a splendid business in diseases of the eye. Failing in health he removed to Texas and located at Dallas, where he has resided continually till his death. Dr. Pope was a courteous gentleman of distinguished appearance and manners, and a skillful surgeon and oculist. His son, Dr. Boling Pope, Jr., and stepson, Dr. Ayers, are connected—one with the Charity Hospital and the other with the Tulane University.

---

**The Professors.**—Prof. A. J. Smith, M. D., Texas Medical College, and of the editorial staff of the JOURNAL, is spending a few weeks at San Antonio with his family. We call attention to his "Note to the Profession" elsewhere in this issue.

Prof. Wm. Keiller, M. D., the celebrated Anatomist, holding the chair of Anatomy in the Texas Medical College, also of the JOURNAL'S staff, is summering this year at San Marcos, the loveliest village in Texas,—made famous by Mollie E. Moore's "Minding the Gap."

Prof. Seth M. Morris, M. D., the Chemist of the Texas Medical College Faculty, as we announced in our last, has taken unto himself a rib, and has gone to Europe. Be back in time to resume labor in the College in September.

Prof. A. G. Clopton, M. D., the Physiologist of the Texas Medical College Faculty, after spending a few weeks in Dallas, has gone traveling north with his family.

And Prof. Cerna—the "new remedy" man—we have forgotten just where he has gone, but—you may depend upon it, you will hear from him on the subject of some "new remedy"—of the interminable "coal-tar-series—with a hard name—when he comes back.

Nor do we remember exactly the whereabouts of the other Professors.—Prof. Thompson, the Surgeon of the Faculty, has gone, as usual—we believe—to Manchester, England, for the summer.

Professors Paine, West, Randall and Kennedy, we believe, remain in Galveston.

**A Word With You Doctor.**—During the period of depression and financial stringency, the management of this JOURNAL have forborne to send its bills for subscription, appreciating the fact that with many of its friends it would be inconvenient to pay. But it is not the case with all; some, we know, are prepared, willing, even anxious to pay, and some have remitted without being asked; others have inquired why they were not notified, etc. Now, we have no means of discriminating, of knowing who can and who cannot pay, and hence we have had to do as best we could without collecting for subscriptions, all this year; still promptly paying our printer for each issue of the JOURNAL. We feel that we must recoup; the longest purse has a bottom and we've n-e-a-r-ly struck the bottom of ours (which is not long). Hence these few lines are to say "to all whom it may concern:" Your bill will be mailed you simultaneously with this (every bill on the book has been made out and sent), and if you have any money and can do so without inconvenience, *divide with us*. Make an effort to catch up. Most of our subscribers are in arrears, for the reasons stated; we have been too considerate of them on account of hard times, to even send statement, and—don't mention it—some (who can pay we know) are away behind, two years and over. This is hardly creditable to a man who wants to pay. Now, let no one especially consider himself dunned; but *it takes money to run a bang up, red hot, first class medical journal*, and we need all the help we can get. Please remit.

N. B. As usual bills include the coming year—as subscription is payable in advance. Part payment will be thankfully received.

---

### RETROSPECTIVE: PROSPECTIVE.

(THE JOURNAL.)

Nine years ago to-day (July 10) Vol. I, No. 1, of the "Red Back," as it is familiarly called, was sent to a few readers; nine years ago to-day DANIEL'S TEXAS MEDICAL JOURNAL unfurled its banner, set sail, and drifted out upon the sea of uncertainty,—a little forty-four-page 5x8 pamphlet, but as full of fire and ambition as the business end of a hornet. What it lacked in size it made up in noise. It attracted attention,—it took; it seemed to "fill a long-felt want." It met the requirements of the time and occasion, for, closely following the *prospectus*, subscriptions came rattling in, in advance, and they have continued to do so, but with a decided let up this year, because of the times.

The splendid support accorded us from the start, the evidences of high appreciation of an earnest and sustained endeavor to establish in Texas a high class and representative medical publication,—something the State had not before had, was as unexpected, though ardently hoped for, as it was gratifying. The JOURNAL at once took position in the foremost rank, a position it has steadily held, strengthened year after year by an appreciative constituency, till now its fame is—well, like the 4th July orator said about the eagle's tail,—"spread *away* out!" It has gone up head, "sure enough," and is not going to be turned down by any strike, adversity, financial stringency, repeal of the Sherman act—passage of the tariff bill, the annexation of the Sandwich Islands, and consequent decline in the price of sandwiches,—nor by any other fellow; we have weathered the storms of 1892-3—and half of '94—the toughest yet encountered, and find ourselves, "tho' slightly disfigured," still on deck, and in ship-shape.

Well—like the tadpole—when he no longer needed the terminal appendage in his business, dropped it, the JOURNAL developing rapidly under stimulus of increased patronage, dropped the prefix "Daniel's," and came out like Mark Tapley—"strong," and (unlike him, however,) in a new dress—a hundred page magazine 6½x10, as red as ever, and as full of vim, vigor and determination, flying the banner, TEXAS MEDICAL JOURNAL [and you bet, *in hoc signo vinces!*]—and here we are. Nine years have rolled around; we are "nine going on ten," and here and now we make our best annual bow—; returning "thanks for past favors and soliciting a continuation of the same," in the language of the shopkeepers.

The JOURNAL outgrew the physical capacity of its founder, and help was needed. The most casual observer cannot fail to have noticed the result of the infusion of new energy into the business; the improvement speaks for itself. The advertising space has doubled in value, while, what is known as "preferred space," is taken in advance, and is at a preminm. We lead in circulation, and recognize no competitor; the JOURNAL covers the ground—and fills the demands.

So—we make our bow,—kiss our hands—wave our handkerchief and—with a final "take care of yourself," the craft sails out of port on her tenth annual voyage,—and asks your prayers, and—remittances!

VOL. X, number 1, greets you to-day as cheerily, and as confident of your good will and support as *ever*. All aboard!

